# Self-Reported Discrimination, Resilience, and Oral Health-Related Quality of Life Among Older Chinese Americans

**DOI:** 10.1093/geroni/igab046.1598

**Published:** 2021-12-17

**Authors:** Weiyu Mao, Bei Wu, Iris Chi, Wei Yang, XinQi Dong

**Affiliations:** 1 University of Nevada, Reno, Reno, Nevada, United States; 2 New York University, New York, New York, United States; 3 University of Southern California, University of Southern California, California, United States; 4 University of Nevada Reno, Reno, Nevada, United States; 5 Rutgers University, Rutgers Institute for Health, New Jersey, United States

## Abstract

There is a lack of empirical evidence on self-reported discrimination and oral health-related quality of life (OHRQoL). Further, the mechanism linking the two constructs is not well understood. This study aimed to examine the relationship between self-reported discrimination and OHRQoL and investigate resilience as a mediator in such a relationship among foreign-born older Chinese Americans. Data came from the Population Study of Chinese Elderly in Chicago collected between 2017 and 2019. The working sample included 3,054 foreign-born Chinese Americans (60+ years of age). Mediation analysis was conducted to examine the direct and indirect pathways towards OHRQoL. Self-reported discrimination was directly and indirectly associated with poorer OHRQoL. Resilience mediated the relationship between self-reported discrimination and OHRQoL. Specifically, individuals experienced discrimination reported weaker resilience, and subsequently, reported poorer OHRQoL. Findings illustrate the importance of studying self-reported discrimination in relation to OHRQoL and further identify resilience as an intermediary pathway to promote OHRQoL.

